# Craniofacial and Cervical Morphology Related to Sagittal Spinal Posture in Children and Adolescents 

**DOI:** 10.1155/2014/638238

**Published:** 2014-09-07

**Authors:** Emil Segatto, Angyalka Segatto, Gábor Braunitzer, Christian Kirschneck, Jochen Fanghänel, Gholamreza Danesh, Carsten Lippold

**Affiliations:** ^1^Department of Orthodontics and Pediatric Dentistry, Faculty of Dentistry, University of Szeged, Tisza Lajos Körút 64, Szeged 6720, Hungary; ^2^Department of Oral Surgery, Faculty of Dentistry, University of Szeged, Tisza Lajos Körút 64, Szeged 6720, Hungary; ^3^Department of Orthodontics, University Medical Center Regensburg, Franz-Josef-Strauß-Allee 11, 93053 Regensburg, Germany; ^4^Department of Orthodontics, Dental Clinic of Witten Herdecke, Alfred-Herrhausen-Straße 50, 58448 Witten, Germany; ^5^Department of Orthodontics, University of Muenster, Waldeyerstraße 30, 48149 Muenster, Germany

## Abstract

Studies on the relationship between body posture and craniofacial parameters often focus on the cervical spine. Thus, less attention has been paid to the morphology of the vertebra C2 that serves as both a structural and functional link between the craniofacial area and the other part of the spine. The objective of this study was to assess the relation of craniofacial features to certain morphological and positional characteristics of the cervical vertebrae and the spine during growth. We determined body posture indices for 69 children and adolescents by means of a radiation-free method (rasterstereography). The morphological and positional analysis of the craniofacial area and the cervical vertebrae was based on standardized lateral X-ray cephalograms. Medium to strong correlations were found between body posture, C2 morphology, and craniofacial parameters. We found significant correlations between the C2 dens axis height and maxillary indices as well as between the C2 dens axis inclination and cephalometrical values of the mandibular area. Similarly the correlation between the C2 dens axis inclination and the postural index flèche cervicale was highly significant (*P* < 0.05, *r* = 0.333). These results suggest that morphological features of the odontoid process may serve as valuable predictive markers in interdisciplinary orthopedic-orthodontic diagnostics.

## 1. Introduction

Abnormal body posture has long been known to be a potential cause of various craniofacial orthopedic and orthodontic conditions [1–3]. Postural abnormalities in preadolescence can usually be traced back to pathological curvatures along the spine, which is vitally important for body balance and stability. Pathological curvatures induce the formation of compensatory curvatures elsewhere along the spine which may result in compensatory head posture [4]. Nonphysiological curvatures in the frontal axis may cause tilting of the head to either side, whereas curvatures in the sagittal axis may result in forward or backward tilts. This problem has been widely discussed in the literature [1, 2, 5]. The introduction of body posture indices came as a result of the development and widespread use of radiation-free methods. A particularly precise postural assessment method is photogrammetry-based computer-aided rasterstereography [6]. This method is popular and widely used in studies investigating the etiology of craniofacial deviations. Most of these studies, however, have concentrated on a limited number of cephalometric indices [7–9]. Alterations of the cervical spine have long been held responsible for compensatory head postures, and their exact causes have been investigated for many years [10]. Early investigations focused on the correlation between the morphological properties of the vertebra C1 (*atlas*) and certain measurements obtained in a natural head position from an X-ray cephalogram [11].

As the first cervical vertebra to form a single joint with the next vertebra (C3), the* axis* (C2) has a particular static and functional role in supporting the skull. The atlantoaxial joint is a complex joint that is not found anywhere else along the spine. Therefore, C2 may be regarded as the first vertebra to link the skull and the* atlas* to the other part of the spine in a regular manner. In spite of this unique position, relatively little attention has been paid to the morphological properties of C2 and their relation to various cephalometrical and postural parameters [12].

In this study, we investigated the relationship between sagittal postural parameters and the results of a cephalometrical examination involving a wide range of cephalometrical indices. We were particularly interested in how the morphological and positional properties of the cervical part of the spine are related to the various cephalometrical parameters. We also paid special attention to the morphological and positional parameters of C2.

## 2. Materials and Methods

### 2.1. Study Subjects

Participants had been retrospectively recruited from a group of initially 100 patients classified as requiring orthodontic treatment within a period of 3 months. Exclusion criteria were presence of orthopedic illness (e.g., idiopathic scoliosis or* Morbus Scheuermann*), orthodontic treatment (ongoing or preceding), fewer than two erupted first molars (both maxilla and mandible), fewer than four erupted incisors, lack of tooth germs, and permanent edentulism. Application of these criteria yielded a final study group of 69 children and adolescents.

The mean patient age was 11 years and 10 months (range: 7 years and 11 months to 16 years and 11 months; SD: 2 years and 2 months). The male to female ratio was 21 : 48. Parents had been informed about the exclusion criteria, the aims, and the procedures of the study in both oral and written form. Underage children and adolescents participated in the study with their parents' informed consent. The study conformed in all respects to the tenets of the Declaration of Helsinki and was approved by the local Ethics Committee.

### 2.2. Cephalometry

Cephalometrical analyses in this study were carried out on the basis of standardized initial orthodontic treatment records. We used lateral skull radiographs for planning the orthodontic treatment of individual patients. In this way, patients were not exposed to additional X-rays for study purposes. All cephalograms were taken digitally by the same operator with a Sirona^©^ Orthophos XGPlus cephalometrical device (Sirona^©^ Dental Systems GmbH, Bensheim, Germany) and set to the program C3F with an image field of 24 × 27 cm. The X-ray source had a focus of 0.5 mm, and the exposure data were 73 kV and 15 mA for 14.9 s. We achieved a total enlargement rate of 11.7% by using a fixed focal plane length of 171.4 cm and a fixed midsagittal plane length of 20 cm and used a reference ruler for exact calibration. Cephalograms were taken in the morning in a natural body position, the so-called orthoposition [13, 14].

**Figure 1:**
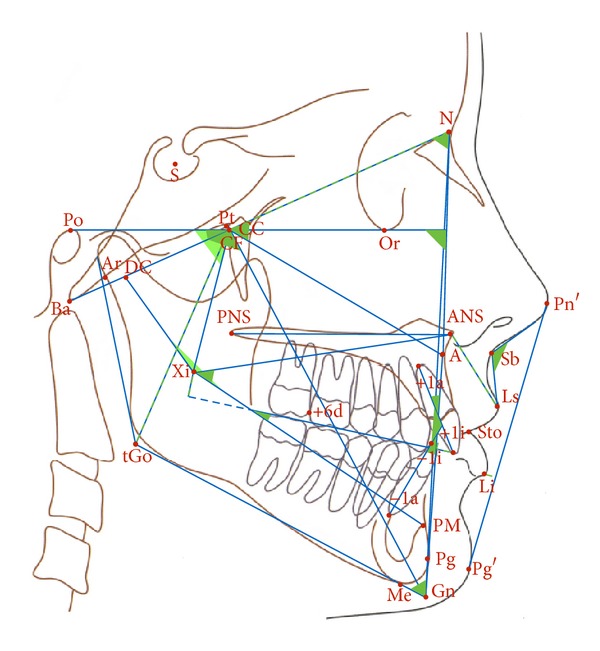
Cephalometric drawing: reference points and measurements.

Cephalograms were analyzed with the Planmeca Romexis Cephalometrical Analysis software 3.0 according to the Ricketts norms [15]. Parameters of craniofacial morphology measured were cranial deflection, facial depth, facial axis, facial taper, and anterior cranial length. To characterize the maxillomandibular complex, we determined lower facial height, Xi-PM/Occ., +1/A-Pg, −1/A-Pg, the interincisal angle, overjet, and overbite. For assessing the maxilla we used the Landes angle, maxillary height, and palatal plane to FH and ramus Xi position, ramus height, and the mandibular arc for defining the mandible. Lip protrusion, upper lip length, and the nasolabial angle were included as indices for facial esthetics (Figure 1).

**Figure 2:**
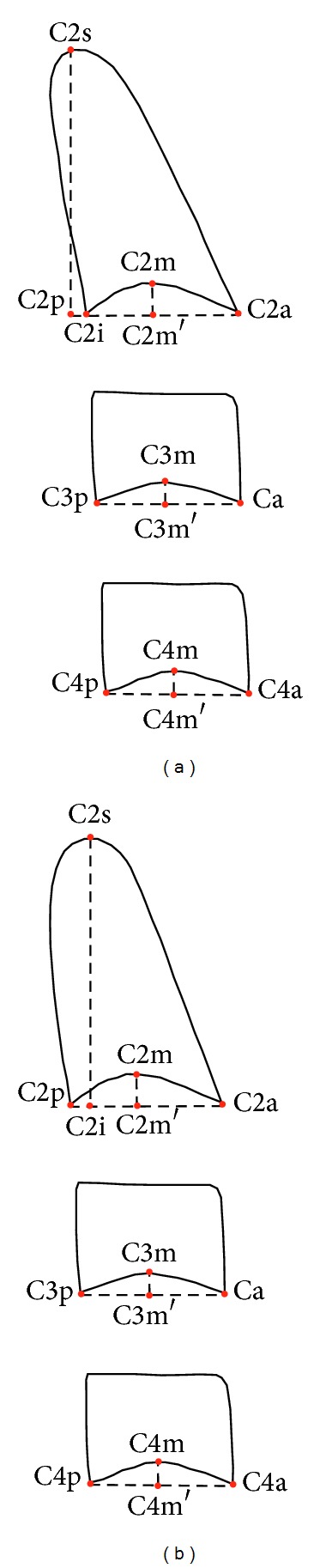
Inclination of the dens axis: (a) posterior inclination; (b) anterior inclination.

The morphology of the cervical vertebrae was described by the following parameters (Figure 2):

- C2p_C2a: the lower P-A width of the body of C2; C2m_C2m′—the lower concavity of the body of C2;
- C2s_C2i: the distance of the apex of C2 from the C2p_C2a line that determines the lower edge of the body of the vertebra;
- C2i_C2p: the posterior distance of the apex of C2 from C2p;
- C2p_C2i: the anterior distance of the apex of C2 from C2p;
- C3p_C3a: the lower P-A width of the body of C3;
- C3m_C3m′: the lower concavity of the body of C3;
- C4p_C4a: the lower P-A width of the body of C4;
- C4m_C4m′: the lower concavity of the body of C4.

**Figure 3:**
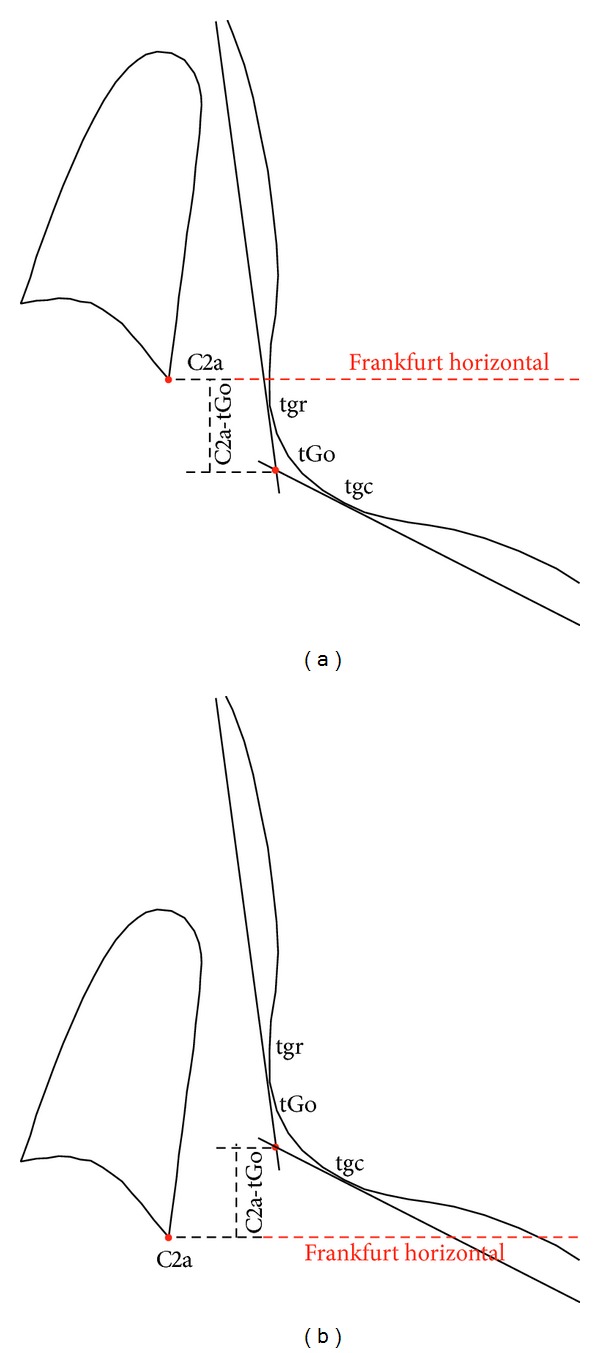
Vertical position of the second vertebra (C2): (a) above tGo; (b) below tGo.

The vertical position of the second cervical vertebra related to the mandibular angle was described by the following parameter (Figure 3):

- C2a_tGo: the vertical distance between the lowermost frontal point of the body of C2 and a plotted point representing the mandibular angle (Gonion, tGo) as measured perpendicularly to the Frankfurt horizontal.

### 2.3. Rasterstereography

**Figure 4:**
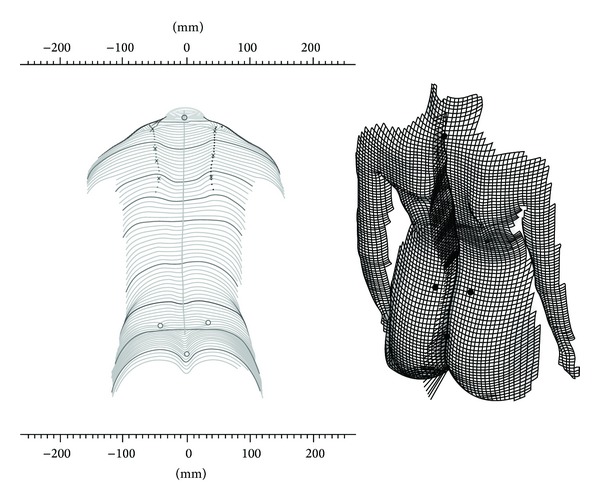
Rasterstereographical back surface reconstruction: an optical line grid is projected onto the back of the patient, while a separate camera compiles optical measurement data from a different direction.

Rasterstereographical images of the backs of the patients were obtained in the same examination session as the lateral skull radiographs with the Formetric II 3D/4D device (Diers International GmbH^©^, Schlangenbad, Germany), which has been designed to generate a three-dimensional photographical image of a person's back in standing position. Images are generated with the help of a fine line grid projected onto the back of the subject. This grid provides information about the surface of the back at an error level of <0.1 mm [16]. Imaging takes 0.04 s. Six sequential images were taken of each patient to reduce the natural sway and breathing effects. Reconstruction of sagittal and frontal sections is made possible by the recognition and software processing of certain significant anatomical structures, such as the iliac spine (SI) and the* vertebra prominens* (VP) [17] (Figure 4). The associated software (Virtual Spine 3.1, Diers^©^) uses mathematical algorithms to construct a 3D model of a person's back. This way, an image of the real deviation of the spine from the vertical axis can be obtained. Sagittal curvatures can be reproduced at a precision of 2.8° [18].

**Figure 5:**
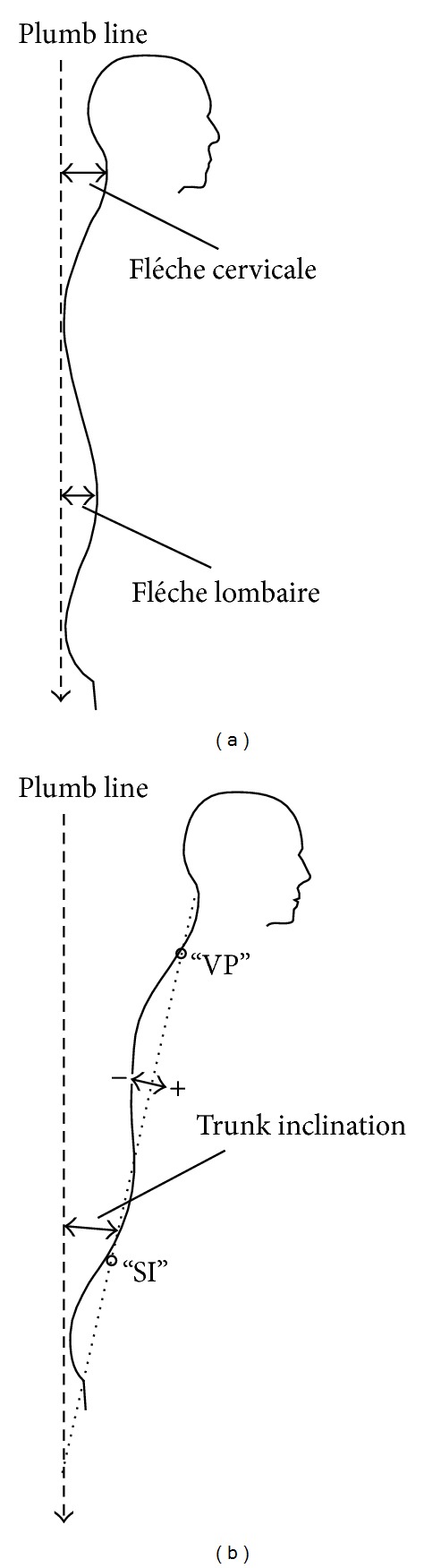
Rasterstereographical measurements (sagittal plane): (a) flèche cervicale or flèche lombaire: sagittal distance between the lowest point of the cervical or lumbar spine and the virtual vertical plumb line; (b) trunk inclination: angle between the connection line of the vertebral point (VP) and the midline of the right (DR) and left (DL) dimple points, representing the* Spina iliaca* (SI) of the upright standing patient toward the virtual vertical plumb line [1].

In the lateral view, sagittal curvatures can be characterized by the indices flèche cervicale and flèche lombaire. These values give the distance of the apex of the cervical and lumbar lordosis from a virtual vertical plumb line [19], yielding a fairly good approximation of the extent of thoracal kyphosis [7] (Figure 5(a)). Body posture, also in the lateral view, is primarily characterized by trunk inclination that is defined as the angle between the real vertical axis and the straight line connecting the midpoints of the lines between the* vertebra prominens* (VP) and the left and right* crista iliaca superior* (SI) (Figure 5(b)) [19].

### 2.4. Statistical Analysis

The cephalometrical and rasterstereographical data of patients were blinded before measurements and statistical analysis. To determine the method error of the cephalometrical measurements, we used the Dahlberg's formula: mean square error *S*_*E*_^2^ = Σ*d* ^2^/2*n* (*d* = difference between repeated measurements; *n* = number of recorded radiographs) [20]. The measurements were repeated on randomly chosen radiographs at 2-week intervals by the same operator. The acceptable error levels were set at 0.5° and 0.5 mm according to Trpkova et al. [21].

All analyses were carried out using the Statistical Package for Social Sciences 17.0 (SPSS Inc., Chicago, Illinois, USA). Descriptive statistics were calculated with regard to mean, standard deviation, and range. Assumptions for parametrical tests were verified prior to significance testing. Correlations between the craniofacial, cervical, and posture parameters were assessed by Pearson's correlation coefficient *r* with *r* > 0.1 denoting a small correlation, *r* > 0.3 a medium correlation, and *r* > 0.5 a strong correlation. The relation of dens axis inclination and trunk inclination was evaluated by means of linear regression analysis. Significance adjustment for multiple comparisons was done with the Šidák correction (general level of significance was set at *P* < 0.05).

## 3. Results

The method error calculated by Dahlberg's formula was below the acceptable reference error levels of 0.5° and 0.5 mm [21] in all instances.

### 3.1. Correlation of Body Posture and Thoracal Kyphosis with Craniofacial Parameters

A correlation analysis of the cephalometrical craniofacial parameters (Table 1) and the rasterstereographical data (Table 3) yielded the following results.

- Trunk inclination was significantly correlated with +1/A-Pg (*P* < 0.05, *r* = −0.284), lip protrusion (*P* < 0.05, *r* = −0.310), anterior cranial length (*P* < 0.05, *r* = 0.249), and ramus height (*P* < 0.05, *r* = 0.305).
- Flèche cervicale was not significantly correlated with any of the variables.
- Flèche lombaire was significantly correlated with the interincisal angle (*P* < 0.05, *r* = −0.275) and lip protrusion (*P* < 0.05, *r* = 0.247).

### 3.2. Correlation of Body Posture and Thoracal Kyphosis with Cervical Parameters

After correlating the cephalometrical cervical parameters (Table 2) with the rasterstereographical data (Table 3) the following results were obtained.

**Figure 6:**
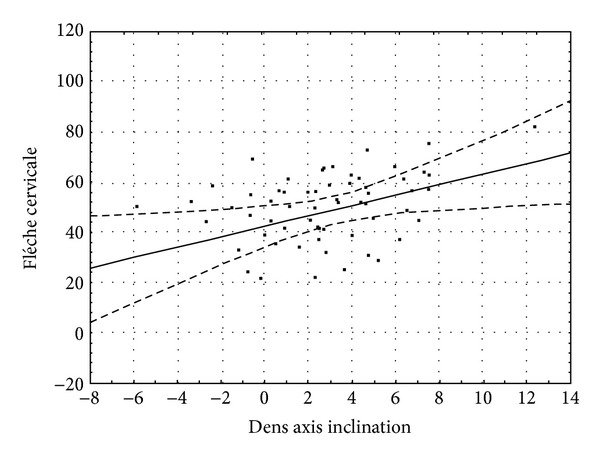
Correlation between dens axis inclination and flèche cervicale. The dashed curves denote the 95% confidence interval.

- Trunk inclination was significantly correlated with the lower concavities of the vertebrae C2 (*P* < 0.05, *r* = 0.453), C3 (*P* < 0.05, *r* = 0.372), and C4 (*P* < 0.05, *r* = 0.393).
- Flèche cervicale was significantly correlated with the lower concavities of the same vertebrae: C2 (*P* < 0.05, *r* = 0.395), C3 (*P* < 0.05, *r* = 0.318), and C4 (*P* < 0.05, *r* = 0.353). In addition, flèche cervicale was significantly correlated with C2i_C2p (*P* < 0.05, *r* = 0.333) (Figure 6).
- Flèche lombaire values were not correlated with any of the cervical vertebrae indices.

**Figure 7:**
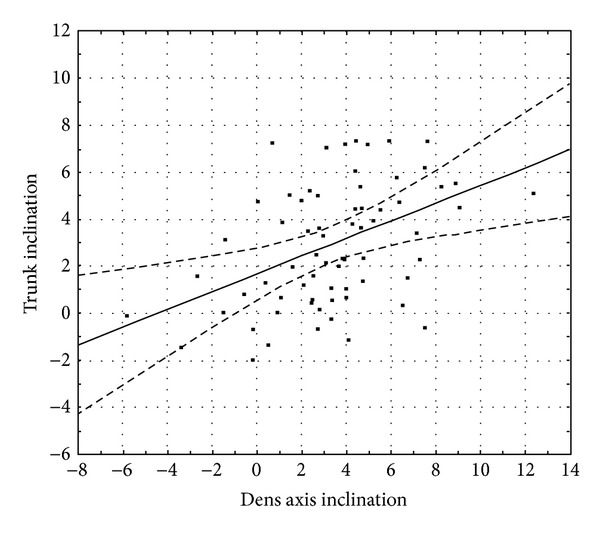
A graphical representation of the results of the regression analysis between dens axis inclination and trunk inclination. The dashed curves denote the 95% confidence interval.

An important aim of this study was to determine whether the projection of the* apex dentis* on the vertebral base (C2i_C2p or C2p_C2i) may be related to any of the rasterstereographical back surface indices. C2i_C2p indicates a projection falling behind the basis of the vertebra, whereas C2p_C2i denotes a projection falling upon the vertebral basis (Figure 2). Therefore, these projections determine forward or backward inclinations. To find out whether an inclination determined in such a manner has affected any of the back surface variables, we carried out a linear regression analysis for the variables dens axis inclination and trunk inclination. We found that trunk inclination significantly predicted dens axis inclination: *β* = 0.31, *t*(67) = 2.66, and *P* < 0.05 (Figure 7).

### 3.3. Correlation of C2 Vertebra Morphology with Craniofacial Parameters

Of the cervical vertebrae indices (Table 2), C2a_tGo (the vertical distance of the body of C2 and the mandibular angle) was significantly correlated with the following craniofacial cephalometrical variables (Table 1): facial axis (*P* < 0.05, *r* = −0.345), facial taper (*P* < 0.05, *r* = −0.408), cranial deflection (*P* < 0.05, *r* = −0.319), Landes angle (*P* < 0.05, *r* = −0.438), ramus height (*P* < 0.05, *r* = −0.478), and −1/A-Pg (*P* < 0.05, *r* = −0.301).

The P-A width of the base of C2 (C2p_C2a) was significantly correlated with upper lip length (*P* < 0.05, *r* = 0.267), facial depth (*P* < 0.05, *r* = 0.289), and ramus height (*P* < 0.05, *r* = 0.327).

The lower concavity of C2 (C2m_C2m′) was significantly correlated with lip protrusion (*P* < 0.05, *r* = −0.296), anterior cranial length (*P* < 0.05, *r* = 0.439), and ramus height (*P* < 0.05, *r* = 0.327).

The height of the dens axis of C2 (C2s_C2i) was significantly correlated with the following cephalometrical indices: upper lip length (*P* < 0.05, *r* = 0.273), maxillary height (*P* < 0.05, *r* = 0.267), palatal plane to FH (*P* < 0.05, *r* = 0.269), anterior cranial length (*P* < 0.05, *r* = 0.335), ramus height (*P* < 0.05, *r* = 0.506), mandibular arc (*P* < 0.05, *r* = −0.264), and −1/A-Pg (*P* < 0.05, *r* = 0.321).

Of the variables characterizing the projection of the* apex dentis* on the basis of the vertebral body, C2i_C2p was significantly correlated with anterior cranial length (*P* < 0.05, *r* = 0.269), whereas C2p_C2i was significantly correlated with the interincisal angle (*P* < 0.05, *r* = 0.800), the nasolabial angle (*P* < 0.05, *r* = −0.695), and +1/A-Pg (*P* < 0.05, *r* = −0.701).

### 3.4. Correlation of C3 Vertebra Morphology with Craniofacial Parameters

The P-A width of the base of C3 (C3p_C3a) (Table 2) was significantly correlated with upper lip length (*P* < 0.05, *r* = 0.277), maxillary height (*P* < 0.05, *r* = 0.238), palatal plane to FH (*P* < 0.05, *r* = 0.262), anterior cranial length (*P* < 0.05, *r* = 0.349), and ramus height (*P* < 0.05, *r* = 0.368) (Table 1), whereas its concavity (C3m_C3m′) showed significant correlation with lip protrusion (*P* < 0.05, *r* = −0.303), anterior cranial length (*P* < 0.05, *r* = 0.269), and ramus height (*P* < 0.05, *r* = 0.321).

### 3.5. Correlation of C4 Vertebra Morphology with Craniofacial Parameters

The P-A width of the body of C4 (C4p_C4a) (Table 2) was significantly correlated with maxillary height (*P* < 0.05, *r* = 0.274) and Xi-PM/Occ. (*P* < 0.05, *r* = 0.299) and its lower concavity (C4 m_C4m′) with lip protrusion (*P* < 0.05, *r* = −0.289), anterior cranial length (*P* < 0.05, *r* = 0.267), ramus height (*P* < 0.05, *r* = 0.449), and +1/A-Pg (*P* < 0.05, *r* = −0.246) (Table 1).

## 4. Discussion

Unlike earlier publications on the subject, we compared the results of rasterstereographical back surface analysis to those of a larger cephalometrical database [19].

The influence of body balance should be discussed regarding the position of the patient positioned meanwhile the examination of the head position and the body balance. The examinations in the lateral cephalographs were done in natural head position. No flexion or extension of the head was performed. The rasterstereographic images of the patients back surface were performed in natural standing position. This results in a normal patient individual body posture and the results of the lateral head cephalographs and the patients data showing kyphosis or lordosis could be analysed.

First of all, our data support the results of earlier craniofacial analyses by providing associated soft tissue and dental indices [7]. For instance, lip protrusion was found to be correlated with trunk inclination and flèche lombaire, whereas +1/A-Pg showed a strong correlation with trunk inclination and the interincisal angle with flèche lombaire.

Although earlier research concentrated primarily on the structures of the dentofacial area, some of the significant but previously not described correlations found in this study may indicate new directions for further research. For instance, the correlation between trunk inclination and anterior cranial length may indicate a link between body posture and the formation of the craniobasal configuration during growth.

None of the cephalometrical indices were significantly correlated with flèche cervicale, and parameters of the cervical vertebrae did not show any significant correlation with flèche lombaire. At the same time, a strong positive correlation was found between the concavity of the bases of C2, C3, and C4, trunk inclination, and flèche cervicale. This correlation verifies that the sagittal curvatures of the spine become accentuated during skeletal maturation.

Beyond the concavity indices, flèche cervicale was also significantly correlated with the posterior projection of the* apex dentis*. The strong positive correlation suggests that the posterior inclination of the dens axis is directly proportional to the extent of kyphosis. As the* apex dentis* takes its final position as early as the age of 7 years [22], the inclination shift is possibly related to the caudal displacement of the lower frontal point of the axis (C2a) that falls exactly on the reference line used for the projection assessment of the* apex dentis* (Figure 2). Such positional changes of the basis of C2 may be traced back to permanent postural irregularities and permanent changes of the spine caused by such irregularities.

The strong correlation between the height of the dens axis with the dentoskeletal parameters of the mandible (e.g., inner gonial angle, lower incisor protrusion) deserves special attention. At the same time, indicators of the inclination of the dens axis show a strong correlation with maxillary parameters, such as the nasolabial angle, the upper incisor inclination, and the interincisal angle.

Therefore, the examined parameters of the cervical vertebrae are primarily correlated with structures of growth during this period.* Dens axis* height and inclination, however, seem to be especially important indices because they show strong correlations with both the sagittal parameters of posture and those structures of the dentofacial area with a highly prognostic value in treatment planning.* Dens axis* height and inclination could thus serve as early predictive markers of dentofacial and posture anomalies.

Earlier studies involving all seven cervical vertebrae failed to find a correlation between cervical curvature and craniofacial morphology in adults [23]. However, such failure may be due to differences in head positions because head positioning has a profound effect on the cervical parameters measured. The results of the present study corroborate the findings of earlier studies of the entire cervical area [24] and indicate the necessity of further measurements that do not require complicated and almost irreproducible X-ray techniques. Although earlier studies have concentrated on the* atlas* (C1), our results suggest that the* dens axis* might also be a promising basis of measurement because its dimensions can be reproducibly determined by means of a cephalogram. If the resolution of the X-ray image is high enough, measurement accuracy may even reach that of CT images [25].

Some significant correlations are difficult to explain. These include the correlation between trunk inclination and lip protrusion or the correlation between dens axis height and upper lip length. We propose that these are not real, generalizable correlations but rather result from the characteristics of our sample.

We also evaluated possible gender differences for the parameters studied, but none were found. Given that the age ranges included the pubertal growth period, this is a counterintuitive result. However, it must be noted that our sample used was not balanced in terms of gender (more than twice as many girls as boys) and that this could account for the gender indifference observed.

## 5. Conclusion

Our measurements of children and adolescents showed new associations between sagittal back surface parameters and a large number of craniofacial indices. Since these measurements were made in children and adolescents during the growth phase, the repetition of such measurements in adults, whose skeletal development is completed, seems to be indicated. Our results suggest that the morphological parameters of the vertebra C2 that is situated at the border of the craniofacial area in a position distinguished in both a structural and functional respect could be efficiently used in interdisciplinary orthopedic-orthodontic diagnostics.

## Figures and Tables

**Table 1:** Descriptive statistics of the cephalometrical analysis of the craniofacial parameters. SD: standard deviation.

Craniofacial parameters	Mean	SD	Minimum	Maximum
Craniofacial Morphology				
Cranial deflection (°)	27.79	2.53	20.77	36.22
Facial depth (°)	85.86	3.08	79.63	92.26
Facial axis (°)	88.54	4.31	76.41	96.52
Facial taper (°)	68.72	4.16	58.58	80.61
Anterior cranial length (mm)	54.28	2.87	48.38	62.40
Maxillomandibular Complex				
Lower facial height (°)	46.46	4.91	33.51	59.58
Xi-PM/Occ. (°)	25.27	3.83	17.01	33.65
Xi-Occ. (mm)	0.75	2.92	−5.86	7.55
+1/A-Pg (°)	28.04	7.54	13.99	46.20
−1/A-Pg (°)	21.59	5.65	9.73	32.50
Interincisal angle (°)	130.37	9.89	112.91	151.76
Overjet (mm)	4.68	2.38	0.30	13.14
Overbite (mm)	2.81	2.17	−2.01	8.21
Maxilla				
Landes angle (°)	60.91	3.20	52.24	67.25
Maxillary height (°)	56.37	3.36	48.32	66.21
Palatal plane to FH (°)	−1.37	3.85	−11.76	10.74
Mandible				
Ramus Xi position (°)	70.11	6.03	39.07	84.01
Ramus height (mm)	57.25	6.06	46.23	70.76
Mandibular arc (°)	152.19	7.96	128.65	170.40
Esthetic relations				
Lip protrusion (mm)	−0.71	2.74	−9.10	4.83
Upper lip length (mm)	20.21	2.06	17.01	26.65
Nasolabial angle (°)	115.11	10.35	79.58	133.31

**Table 2:** Descriptive statistics of the cephalometrical analysis of the cervical parameters. SD: standard deviation.

Cervical parameters	Mean	SD	Minimum	Maximum
Cervical vertebra morphology				
C2a_tGo (mm)	4.41	4.43	−4.02	14.77
C2p_C2a (mm)	12.42	1.33	8.68	15.26
C2m_C2m′ (mm)	0.71	0.59	0.00	2.42
C2s_C2i (mm)	30.20	2.40	24.25	36.66
C2i_C2p (mm)	3.95	2.38	0.00	12.34
C2p_C2i (mm)	2.07	1.79	0.19	5.86
C3p_C3a (mm)	12.65	1.26	8.75	17.06
C3m_C3m′ (mm)	0.62	0.54	0.00	2.21
C4p_C4a (mm)	12.66	1.24	8.69	16.62
C4m_C4m′ (mm)	0.43	0.46	0.00	1.99

**Table 3:** Descriptive statistics of the rasterstereographical analysis. SD: standard deviation.

Rasterstereographical sagittal values	Mean	SD	Minimum	Maximum
Flèche cervicale (mm)	48.58	23.71	0.00	102.80
Flèche lombaire (mm)	29.32	12.87	5.31	54.80
Trunk inclination (°)	2.82	3.37	−3.96	11.09
